# European Society for Vascular Surgery (ESVS) 2020 clinical practice guidelines on the management of acute limb ischaemia; a word of caution!

**DOI:** 10.1186/s42155-020-00122-5

**Published:** 2020-05-18

**Authors:** M. Hamady, S. Müller-Hülsbeck

**Affiliations:** 1 Department of Interventional Radiology, praed street, Imperial College-London, London, W2 1NY UK; 2 Department of Diagnostic and Interventional Radiology/Neuroradiology, Ev.-Luth. Diakonissenanstalt zu Flensburg Zentrum für Gesundheit und Diakonie, Knuthstr. 1, 24939 Flensburg, Germany

Acute limb ischemia (ALI) is relatively uncommon but serious condition. The estimated incidence is 23.3/100.000 person years (Baril et al. 2014) (Korabathina et al. 2013). Regardless of any intervention, ALI is linked with significant morbidity and mortality rates. The hospital mortality ranges from 6.3–9% with hospital amputation rate of 6%, and one-year amputation rate 11%, while the one-year mortality could reach up to 41%.

ESVS has published guidelines regarding the management of this critical condition (Björck M et al. 2020). The guidelines have adopted the grading system of recommendation strength proposed by the European Society of Cardiology. In essence, the evidence behind each recommendation is given A-C level, where A represents data derived from multiple randomised clinical trials or meta-analyses, B data derived from a single randomised clinical trial or large nonrandomised studies and C consensus of opinion of the experts and/or small studies, retrospective studies and registries. The strength of each recommendation is given I to III class level where class I represents evidence and/or general agreement that a given treatment or procedure is beneficial, useful, and effective, Class II conflicting evidence and/or a divergence of opinion about the usefulness/efficacy of the given treatment or procedure, and Class III evidence or general agreement that the given treatment or procedure is not useful/effective, and in some cases may be harmful.

Sixty-one recommendations have been proposed, covering various aspects of ALI including epidemiology, clinical presentations, diagnosis and management. Generally speaking, the authors have done excellent effort trying to produce a document that help practitioners to offer best care and aid in decision making process.

The purpose of this editorial is to comment on few specific points, namely the use of what is called “hybrid theatre” and “hybrid treatment” of ALI.

Hybrid theatre is a vague term with no clear or agreed definition. It has been frequently used to serve certain political agenda. The best and only respected definition of the term “hybrid theatre” comes from the Medicine and Health Regulatory Agency of United Kingdom (Medicines and Health Products Regulatory Agency (MHRA) 2010), following collaborative work from MHRA, British Society of Interventional Radiology, Royal College of Radiologists, and Vascular Society of Great Britain and Ireland. It states that the facility offering endovascular aortic repair should encompass beside best imaging equipment, theatre specifications including proper ventilation, lights, resuscitation trolleys, infection control measures, equipment stock and backup facility. There is no agreed specific area of the foot print, but area ranges from 45 to 58 m^2^ thought to be adequate. In that sense almost all modern angiosuites in the Western World have these criteria and can be called “hybrid” theatres. The question of cost effectiveness of location and multipurpose use of “hybrid” theatre is much more controversial and beyond the scope of this editorial (Fletcher et al. 2017) (Field et al. 2009).

What about the term “hybrid treatment”?. Recommendation 14 and related discussion states “that patients with acute limb ischaemia should have access to treatment in a hybrid theatre/ operating theatre with C arm equipment, and by a clinical team able to offer a full range of open or endovascular interventions during a single procedure”. It is obvious that this recommendation is not supported by any credible evidence and the authors are right to give it Level C reflecting the political consensus behind this recommendation.

First of all, there is no clarity about the term “hybrid treatment”. It is once used to refer to combination of adjuvant technique to thrombolysis such as stent, angioplasty and once to combined endovascular and open revascularisation. The guidelines make an assumption that combined endovascular and thromboembolectomy can be useful to treat some patients with residual stenosis or thrombus in one setting. The authors admit that there is no data to support this recommendation and it is more of a political consensus rather than science. In support for this particular recommendation, the guidelines referenced mainly three small, single centre and retrospective studies. Beside several limitations and flaws in each one of those papers, the shared finding is the lack of significant difference in amputation rate or survival. There is no count of cost, duration of index procedure nor direct comparison between several intervention approaches (de Donato et al. 2014; Balaz et al. 2013; Argyriou et al. 2014). In a valuable study of 1480 patients with ALI treated at 45 centres in USA which is quoted in the guidelines document, no significant benefit of “hybrid procedure” is proven (Davis et al. 2018). In this multicentre study, endovascular repair actually shows better short term results than open or hybrid repair in terms of amputation rate. The study does show no difference in reintervention rate or survival to favour hybrid repair.

The other controversial point is related to recommendation 18. The guidelines recommend the use of table angiography in each patient undergoing open or endovascular revascularisation. However, the evidence to support completion angiography in every case is lacking. A simple hand hold CDUS (coloured duplex ultrasound), Ankle-Brachial Index Pressure and inspection of the foot are simple, much more cost effective and less physiologically harmful to the patient than full DSA angiography. This particular recommendation is based on two weak references (Lipsitz and Veith 2001) (Zaraca et al. 2010).

In fact, one of those two references is an editorial type paper in 2001 and the second one is a retrospective study of 384 patients. The latter reference divides the relatively small patients’ cohort into two groups; one with and one without completion angiography. The authors conclude that there are no differences in amputation rate or survival outcome between the two groups.

Last but not least, recommendations 24 and 25 state that “(percutaneous) catheter-directed thrombolysis is considered (Rutherford grade IIa) or may be considered (Rutherford grade IIb)” for ALI treatment. We agree that there is a signal to suggest that revascularisation can be accelerated by using combined mechanical thrombectomy with thrombolysis which could be supplemented by angioplasty with or without stent. In a study of 79 patients with acute arterial occlusion, restoration of blood flow is achieved in 77% following total endovascular approach with no surgical conversion (Saxon et al. 2018). The use of mechanical thrombectomy as adjunct to thrombolysis is promising and needs further investigation and larger studies to assess the efficacy and cost effectiveness. Recommendation 24 and 25 could have been a little bit sharper and precise in order to indicate catheter-directed thrombolysis more as a first-line treatment instead of considering it as an alternative.

Finally, it must be made clear that guidelines are general opinion and should not be considered as didactic instructions to practice. Final intervention approach depends on local factors, particular patient’s condition and multidisciplinary discussion and decision to offer this dedicated patient group ideal and individualized treatment.

## Data Availability

Not applicable.
